# HPV vaccine behaviors and intentions among a diverse sample of women aged 27-45 years: implications for shared clinical decision-making

**DOI:** 10.1186/s12889-024-18740-2

**Published:** 2024-08-08

**Authors:** Jennifer D. Allen, Nadia N. Abuelezam, Raviv Rose, Katelin Isakoff, Gregory Zimet, Holly B. Fontenot

**Affiliations:** 1 Department of Community Health, Tufts University School of Arts and Sciences, 574 Boston Ave, Medford, MA 02155 USA; 2 Connell School of Nursing, Boston College, 140 Commonwealth Ave., Chestnut Hill, MA 02467 USA; 3 Department of Pediatrics, Division of Adolescent Medicine, School of Medicine, Indiana University, 410 W. 10th Street, HS 1001, Indianapolis, IN 46202 USA; 4 Nancy Atmospera-Walch School of Nursing, University of Hawaii at Manoa, 2528 McCarthy Mall, Honolulu, HI 96822 USA

**Keywords:** Human papillomavirus, Adult women, HPV vaccine, Attitudes and behaviors

## Abstract

**Background:**

The Advisory Committee on Immunization Practices issued a shared clinical decision-making (SCDM) recommendation for HPV vaccination in persons aged 27–45. Since expanded eligibility for the vaccine was issued, little information has been available about HPV vaccine behaviors and intentions among women in this age group.

**Methods:**

We conducted a cross-sectional online survey among women aged 27–45 years recruited through a Qualtrics™ respondent panel (*N* = 324) to answer the following questions (1) What is the prevalence of HPV vaccination among a diverse sample of adult women aged 27–45 years? (2) What are the characteristics of those who have or have not previously been vaccinated? and (3) What factors are associated with the intention to obtain the HPV vaccine among those who had never been vaccinated? Multivariable logistic regression analyses estimated adjusted odds ratios (AORs) and 95% confidence intervals (95% CIs).

**Results:**

Only 31.1% had at least one dose of the HPV vaccine. In multivariable analyses, those more likely to have been vaccinated were younger and were more likely to believe that the vaccine was effective. Of those unvaccinated or unsure, 54.8% indicated they were likely to get vaccinated in the future. Factors associated with future vaccine intention (compared to those not intending) included beliefs about vaccine testing, perceived likelihood of HPV infection, greater comfort in asking one’s provider for vaccination, and prior negative healthcare experiences.

**Conclusions:**

Our findings suggest that many women in this age group are interested in HPV vaccination. While the recommendation is for SCDM rather than routine vaccination for all women in this age group, efforts to promote informed decision-making among mid-adult women may include educating women about the rigorous vaccine testing and approval process, their risk factors for HPV infection, and encouraging them to engage in SCDM with their medical providers. Targeted efforts to reach women who have had negative experiences with healthcare may also be needed.

**Supplementary Information:**

The online version contains supplementary material available at 10.1186/s12889-024-18740-2.

## Introduction

Despite the availability of primary prevention via vaccination, the human papillomavirus (HPV) remains the most common sexually transmitted infection in the United States (U.S.). Each year, approximately 14 million people in the U.S. are newly infected with HPV, and about 35,900 develop a new cervical or other HPV-related cancer [1, 2]. Highly effective HPV vaccines have been available since 2006 and, until recently, were only approved for those aged 9 through 26 years by the U.S. Food and Drug Administration (FDA). In 2018, the FDA expanded its approval to include persons aged 27–45 years (“mid-adults”) [3]. Subsequently, in 2019, the Advisory Committee on Immunization Practices (ACIP) issued a shared clinical decision-making recommendation for HPV vaccination for mid-adults not previously vaccinated [4]. SCDM involves a discussion between patients and providers about the best available evidence for HPV vaccination, individual risks, and vaccine benefits versus potential harms to arrive at a mutually agreeable decision [5]. As this recommendation differs from routine vaccination that is recommended for younger women, there is a need to understand the extent to which the population of mid-adult women not previously vaccinated would prefer to be vaccinated with the knowledge that they are now potentially eligible [6].

While expansion of eligibility for the HPV vaccine among persons aged 27–45 years provides a significant step towards minimizing the potential burden of HPV-related cancers, uptake of the vaccine among populations for whom it had been previously recommended (routine recommendation for adolescents aged 11–13 years; catch up recommendation for those aged 18–26 years) has yet to meet the national goal of 80% series completion [7]. National data from 2018 show that only 65% of adolescents and 21.5% of women aged 18–26 had completed the recommended vaccine series [8, 9]. Women not previously vaccinated but beyond the (previous) age limit of 26 years are now eligible for HPV vaccination. Despite the plethora of literature explicating factors associated with HPV vaccine uptake in general [10–13], there is limited research examining predictors among women ages 27–45 years, which may inform the SCDM process.

Our study was guided by tenets of the Theory of Reasoned Action, which postulates that behavioral intention is the strongest predictor of future behavior, barring environmental constraints. Intention, in turn, is shaped by attitudes, beliefs, subjective norms, perceived behavioral control, and environmental factors [14]. In this study, we examined attitudes and beliefs about vaccines (e.g., safety, efficacy), as well as the perceived risk of HPV infection and cervical cancer. For social influences, we examined perceptions of social pressure to be vaccinated, including the extent to which significant others (e.g., spouse, friends) support vaccination, and characteristics of the patient/provider relationship (e.g., trust, comfort). We assessed self-efficacy about requesting information or vaccination from one’s provider. We also evaluated environmental barriers, including vaccine cost and prior negative experiences with the healthcare system.

The objectives of this study were to answer three research questions (RQs): (1) What is the prevalence of HPV vaccination among a diverse sample of mid-adult women aged 27–45 years? (2) What are the characteristics of those who have or have not previously been vaccinated? and (3) What factors are associated with the intention to obtain the HPV vaccine among those who have never been vaccinated? Findings will guide healthcare provider vaccine communication strategies and efforts to promote SCDM about HPV vaccination among adult women.

## Materials and methods

### Participants

We conducted an online survey of adult women between April 13, 2020, and June 8, 2020. Participants were drawn from the Qualtrics^XM^ Panel, a database of potential online survey participants maintained by Qualtrics,^XM^ and all respondents have already agreed to be contacted for research purposes. Criteria for inclusion were persons assigned female at birth, aged 27 to 45 years, resident of the U.S., and ability to read and understand English. Given documented differences in HPV vaccination across racial/ethnic groups [15], we oversampled women of minoritized racial/ethnic groups to produce a sample that was 25% Black, 25% Asian, and 25% Hispanic. A further sampling quota was set to recruit a sample that included 25% of respondents who identified as a sexual minority (e.g., bisexual, lesbian, queer, other). Study consent procedures were conducted electronically before the start of the survey (by clicking on a box indicating consent), participation was voluntary, and the standard Qualtrics^XM^ remuneration ($6) was provided. This study was administered online and approved by Tufts University Institutional Review Board.

### Measures

We utilized a number of existing measures on the survey (see supplementary file). First, we assessed both prior receipt and future intentions to get the HPV vaccine, as well as key independent variables from our conceptual framework (i.e., attitudes/beliefs, social influences, self-efficacy, and barriers), as described below. All participants responded to questions related to demographics, vaccination status, and attitudes/beliefs toward HPV vaccination. Only those who reported never having had the HPV vaccine were asked about their future intentions to get vaccinated, and questions were asked to assess social influences, self-efficacy, and barriers toward future HPV vaccination.

*Receipt of and intention to vaccinate*. Using items from the Behavioral Risk Factor Surveillance System [16], we asked participants if they had ever received the HPV vaccine (yes, no or unsure). Those who had not been vaccinated or were unsure were asked, “How likely are you to get the HPV vaccine?” with responses on a 4-point Likert scale (“very likely” to “very unlikely”).

*Attitudes and beliefs*. To assess attitudes and beliefs about vaccines, we asked: [1] “Do you think vaccines are well tested before being made available to the public?” (yes, no, don’t know) [2], “In your opinion, how safe is the HPV vaccine?” (4-point Likert: “very safe” to “not at all safe”/ “don’t know” with higher scores indicating greater perceived safety) [3], “In your opinion, how effective is the HPV vaccine?” (4-point Likert, “very effective” to “not at all effective”/ “don’t know,” with higher scores indicating greater perceived effectiveness), and [4] “If you were making a decision about getting the HPV vaccine, how much would potential side effects influence your decision?” (4-point Likert, “a great deal” to “not at all,” with higher scores indicating greater concern about side effects). To assess perceived susceptibility to cervical cancer and HPV infection, we asked [1] “Compared to the average person your age, would you say you are” (“more likely to get cervical cancer,” “as likely to get cervical cancer,” “less likely to get cervical cancer”); and [2] “How likely are you to get HPV?” (4-point Likert, “very likely” to “very unlikely”/ “unsure”).

*Social influences.* To assess social factors that influence decision-making about getting the HPV vaccine, we asked, “How much would each of the following factors influence your decision?” Response options included: whether you have a spouse or long-term monogamous partner; how other people close to you might think about your decision; comfort and/or trust with [my] health care provider; and comfort and trust in seeking health care (4-point Likert, “a great deal” to “not at all” for each).

*Self-efficacy.* We assessed comfort with engaging in SCDM with health care providers by asking two items: [1] “How comfortable would you be asking your health care provider to give your information regarding the HPV vaccine?” and [2] “How comfortable would you be asking your health care provider to give you the HPV vaccine?” (4-point Likert, “very comfortable” to “very uncomfortable”/ “unsure/don’t know”).

*Barriers.* We assessed the extent to which cost and prior negative experiences with the healthcare system impacted vaccine decisions. Specifically, we asked: “If you were making a decision about getting the HPV vaccine, how much would each of the following factors influence your decision?” The factors listed were cost, insurance coverage, and previous negative health care experiences with response options on a 4-point Likert scale (“a great deal” to “not at all”).

*Demographic Characteristics.* Demographic characteristics were assessed using standard items from the Behavioral Risk Factors Surveillance Survey [16] and included: race/ethnicity [non-Hispanic (NH) White, NH Black, NH Asian, Hispanic and multi-racial]; age (27–29, 30–39, 40–49); income (<$34,000, $35–74,000, >$75,000, not sure); education (high school or less, college or some college, graduate degree); employment (employed, unemployed); and insurance status (public, private, none). We also assessed prior history of cervical cancer screening [1] “Have you ever had an abnormal Pap test?; [2] Have you ever had a positive HPV test?”; and 3) Have you ever been told by a healthcare provider that you had cervical cancer?” (“yes”, “no”, don’t know”).

### Analysis

Our analytic goals were to describe the prevalence of HPV vaccination among mid-adult women (RQ #1), identify factors associated with vaccination status (RQ #2), and examine factors associated with the intention to be vaccinated for HPV among those who had never been vaccinated (RQ #3). Descriptive statistics were tabulated for the total sample, previously vaccinated individuals (≥ 1 dose), and unvaccinated individuals (RQ #1). For RQ #2, we examined factors associated with already having received ≥ 1 dose of the HPV vaccine versus not having received any doses. For RQ #3, we focused only on those who had *not* received any doses of the HPV vaccine. For this analysis, we examined factors associated with falling into two categories: (1) those who report being very likely or likely to get the HPV vaccine and (2) those who report being very unlikely or unlikely to get the vaccine.

The data analysis was completed using SPSS Statistics Version 26 (IBM Corp., Armonk, NY). For RQs #2 and #3, Chi-squared tests were used to assess bivariate associations. For both RQ #2 and RQ #3, univariable logistic regression models were first run with each of the covariates. Then, stepwise selection was used in the multiple logistic regression analyses (slentry = 0.20 and slstay = 0.10); all covariates with association of *p* < 0.10 were included, and *p* *≤* 0.05 was considered statistically significant in the final models. For the multivariable analyses, models were run to assess (RQ #2) factors associated with vaccination status and (RQ #3) factors related to intention to be vaccinated for HPV among those who had never been vaccinated. For these models, those who identified as NH Asian and multi-racial were combined due to the small sample sizes.

## Results

### Sample characteristics, HPV vaccination prevalence (RQ#1), and factors associations with HPV vaccination (RQ#2)

A total of 324 women completed the survey. The majority were between the ages of 30 and 39 years (51.2%), identified as heterosexual (73.8%), had some college or a college degree (61.1%), and had health insurance (33.3% public, 51.5% private). Approximately half of the sample was married (46.6%). Due to the use of quota sampling, race/ethnicity was distributed relatively evenly (NH White 25.6; NH Black 23.58%; NH Asian and another race 27.2%, and 23.8% Hispanic). Only 31.1% (*n* = 101) of the sample reported that they had one or more doses of the HPV vaccine, and 68.9% (*n* = 223) reported not being vaccinated or being unsure about vaccination status. See Table 1.

**Table 1:** Sample characteristics, HPV vaccination prevalence (RQ#1), and bivariate associations with HPV vaccination (RQ#2) (*N* = 324)

	Total (*N* = 324)	Vaccinated (> 1 dose) (*N* = 101; 31.1%)	Not vaccinated/Unsure (*N* = 223; 68.9%)	*p*-value*
	*N* (%)	*N* (%)	*N* (Row %)	
**Characteristic**				
Age				*p* < 0.001
27–29	70 (21.6)	35 (50.0)	35 (50.0)	
30–39	166 (51.2)	53 (31.9)	113 (68.1)	
40–45	88 (27.2)	13 (14.8)	75 (85.2)	
Marital Status				*p* = 0.80
Married	151 (46.6)	46 (30.5)	105 (69.5)	
Not Married	173 (53.4)	55 (31.8)	118 (68.2)	
Sexual Orientation				*p* = 0.34
Heterosexual	239 (73.8)	71 (29.7)	168 (70.3)	
Bisexual/Lesbian/Queer/Other	85 (26.2)	30 (35.3)	55 (64.7)	
Race				*p* = 0.30
NH White	83 (25.6)	29 (34.9)	54 (65.1)	
NH Black	76 (23.5)	17 (22.4)	59 (77.6)	
Hispanic	77 (23.8)	26 (33.8)	51 (66.2)	
Asian and Other	88 (27.2)	29 (33.0)	59 (67.0)	
Education				*p* = 0.34
High school or less	53 (16.4)	20 (37.7)	33 (62.3)	
College or some college	198 (61.1)	56 (28.3)	142 (71.7)	
Graduate degree	73 (22.5)	25 (34.2)	48 (65.8)	
Employment				*p* = 0.99
Employed	226 (70.0)	70 (31.0)	156 (69.0)	
Unemployed	97 (30.0)	30 (30.9)	67 (69.1)	
Income				*p* = 0.89
<$35,000	92 (28.4)	29 (31.5)	63 (68.5)	
$35,000-$74,000	116 (35.8)	38 (32.8)	78 (67.2)	
>$74,000	105 (32.4)	30 (28.6)	75 (71.4)	
Not sure	11 (3.4)	4 (36.4)	7 (63.6)	
Insurance status				*p* = 0.047
Not Insured	49 (15.1)	9 (18.4)	40 (81.6)	
Public	108 (33.3)	41 (38.0)	67 (62.0)	
Private	167 (51.5)	51 (30.5)	116 (69.5)	
Ever had a pap test				*p* = 0.36
No/Don’t know	33 (10.2)	8 (24.2)	25 (75.8)	
Yes	291 (89.8)	93 (32.0)	198 (68.0)	
Ever have an abnormal pap				*p* = 0.66
No/Don’t know	196 (66.7)	61 (31.1)	135 (68.9)	
Yes	98 (33.3)	33 (33.7)	65 (66.3)	
Ever had an HPV test				*p* < 0.01
No/Don’t know	156 (48.1)	36 (23.1)	120 (76.9)	
Yes	168 (51.9)	65 (38.7)	103 (61.3)	
Ever told you had cervical cancer				*p* < 0.01
No/Don’t know	304 (93.8)	89 (29.3)	215 (70.7)	
Yes	20 (6.2)	12 (60.0)	8 (40.0)	
**Attitudes/beliefs**				
Believe vaccines are well-tested				*p* = 0.11
No	48 (14.8)	12 (25.0)	36 (75.0)	
Not sure	62 (19.1)	14 (22.6)	48 (77.4)	
Yes	214 (66.0)	75 (35.0)	139 (65.0)	
Perceive HPV vaccine to be safe				*p* < 0.001
Not at all/Not Very/Don’t know	96 (29.6)	13 (13.5)	83 (86.5)	
Very or fairly safe	228 (70.4)	88 (38.6)	140 (61.4)	
Perceive HPV vaccine to be effective				*p* < 0.001
Not very effective or Don’t know	117 (37.1)	17 (14.5)	100 (85.5)	
Very or fairly effective	198 (62.9)	83 (41.9)	115 (58.1)	

**Bold** are *p* < 0.10 and qualify for entry into multivariable analysis

In the bivariate analyses, age (*p* < 0.001), health insurance status (*p* = 0.047), report of ever having had an HPV test (*p* < 0.01), and having been given a diagnosis of cervical cancer (*p* < 0.01) were associated with having had at least one dose of the HPV vaccine. Attitudes and beliefs that were significantly associated with vaccination status included perceived safety of the HPV vaccine (*p* < 0.001), perceived effectiveness of the HPV vaccine (*p* < 0.001), and perceived likelihood of HPV infection (*p* = 0.068) (Table 1).

In the multivariable analysis, those aged 30 years or older had lower odds of receiving the HPV vaccine compared to those aged 27–29 years (AOR = 0.50; 95% CI = 0.26–0.96). Women who expressed stronger belief in vaccine effectiveness had higher odds of being vaccinated than those who reported that they did not view the vaccine as effective or did not know about effectiveness (AOR = 2.75; 95% CI = 1.33–5.71) (Table 2).

**Table 2:** Multivariable Analysis: Factors Associated with HPV Vaccination (RQ#2) (*N* = 324)*

	Adjusted OR (95%CI)
Characteristic*	
Age	
27–29	ref
30–39	0.50 (0.26, 0.96)**
40–45	0.15 (0.07, 0.35)**
Insurance status	
Not insured	ref
Public	2.33 (0.94, 5.77)
Private	1.79 (0.75, 4.30)
Ever had an HPV test	
No/Don’t know	ref
Yes	1.59 (0.91, 2.76)
Ever told you had cervical cancer	
No/Don’t know	ref
Yes	2.23 (0.72, 6.94)
Attitudes/beliefs*	
Perceive HPV vaccine to be safe	
Not at all/Not very/Don’t Know	ref
Very/fairly safe	1.74 (0.78, 3.92)
Perceive HPV vaccine to be effective	
Not at all/Not Very/ Don’t Know	ref
Very/fairly effective	2.75 (1.33, 5.71)**
Perceived likelihood of getting an HPV infection	
No chance/unlikely	ref
Unsure	0.49 (0.24, 1.02)
Certain/likely/moderately likely	0.71 (0.36, 1.43)

*All factors at *p* < 0.10 from bivariate analyses were entered into final model

**Bold are *p* < 0.05

### RQ #3: Factors associated with the intention to obtain the HPV vaccine among those who had never been vaccinated or were unsure

Of the 223 participants who reported not being vaccinated, 6 did not complete the remainder of the survey items, so the final sample size for RQ #3 and RQ #4 was *n* = 217. In bivariate analyses, women who intended to be vaccinated were those who reported having had an abnormal Pap test (*p* < 0.001) or positive HPV test (*p* < 0.05), stronger beliefs that vaccines are well tested (*p* < 0.001), believed that the HPV vaccine was safe (*p* < 0.001) and effective (*p* < 0.001), and perceived themselves to be more likely to get an HPV infection (*p* < 0.05). Furthermore, social influences [i.e., other people’s opinions (*p* = 0.08) and health care provider recommendation (*p* < 0.05)] a self-efficacy [i.e., comfort asking a health care provider for information about HPV vaccine (*p* < 0.01) and HPV vaccination (*p* < 0.001)], were more likely to report the intention to get vaccinated. In contrast, barriers [i.e., reporting that previous negative healthcare experiences would influence vaccine decision-making (*p* = 0.06)] were associated with lower future vaccination intentions (Table 3).

**Table 3:** Bivariate Analysis: Associations related to the likelihood of getting the HPV vaccine in the future (RQ#3) (*N* = 217)

	Very likely/likely to get HPV vaccine (*N* = 119) *N* (Row %)	Very unlikely/unlikely to get HPV vaccine (*N* = 98) *N* (Row %)	*p*-value*
**Characteristic**			
Age			*p* = 0.26
27–29	23 (67.6)	11 (32.4)	
30–39	57 (51.8)	53 (48.2)	
40–49	39 (53.4)	34 (46.6)	
Marital Status			*p* = 0.57
Married	58 (56.9)	44 (43.1)	
Not Married	61 (53.0)	54 (47.0)	
Sexual Orientation			*p* = 0.28
Heterosexual	86 (52.8)	77 (42.2)	
Bisexual/Lesbian/Queer/Other	33 (61.1)	21 (38.9)	
Race			*p* = 0.51
NH White	30 (56.6)	23 (43.4)	
NH Black	26 (46.4)	30 (53.6)	
Hispanic	30 (60.0)	20 (40.0)	
Asian/Other	33 (56.9)	25 (43.1)	
Education			*p* = 0.72
High school or less	19 (61.3)	12 (38.7)	
College or some college	76 (54.3)	64 (45.7)	
Graduate degree	24 (52.2)	22 (47.8)	
Employment			*p* = 0.69
Employed	82 (53.9)	70 (46.1)	
Unemployed	37 (56.9)	28 (43.1)	
Income			*p* = 0.17
<$35,000	40 (65.6)	21 (34.4)	
$35,000-$74,000	38 (50.0)	38 (50.0)	
>$74,000	39 (53.4)	34 (46.6)	
Insurance Status			
Not insured	19 (51.4)	18 (48.6)	*p* = 0.17
Public	42 (64.6)	23 (35.4)	
Private	58 (50.4)	57 (49.6)	
Ever had an abnormal Pap			*p* < 0.01
No/Don’t know	77 (49.7)	76 (50.3)	
Yes	44 (68.8)	20 (31.3)	
Ever had an HPV test			*p* < 0.05
No/Don’t know	92 (51.7)	86 (48.3)	
Yes	27 (69.2)	12 (30.8)	
Ever told you had cervical cancer			*p* = 0.24
No/Don’t know	113 (54.1)	96 (45.9)	
Yes	6 (75.0)	2 (25.0)	
**Attitudes & Beliefs**			
Believe vaccines are well-tested			*p* < 0.001
No/Don’t know	29 (35.8)	52 (64.2)	
Yes	90 (66.2)	46 (33.8)	
Perceive HPV vaccine to be safe			*p* < 0.001
Not at all/Not Very/Don’t know	30 (36.6)	52 (63.4)	
Very or fairly safe	89 (65.9)	46 (34.1)	
Perceive HPV vaccine to be effective			*p* < 0.001
Not very effective or Don’t know	42 (43.3)	55 (56.7)	
Very or fairly effective	74 (66.1)	38 (33.9)	
Perceived likelihood of cervical cancer			*p* = 0.12
Less likely to get	37 (48.1)	40 (51.9)	
About as likely to get	61 (55.5)	49 (44.5)	
More likely to get	21 (70.0)	9 (30.0)	
Perceived likelihood of getting an HPV infection			*p* < 0.05
No chance/unlikely	52 (46.8)	59 (53.2)	
Unsure	29 (55.8)	23 (44.2)	
Certain/likely/moderately likely	38 (70.4)	16 (29.6)	
**Social influences**			
Is your decision to be vaccinated influenced by having a spouse or long-term partner?			*P* = 0.64
Not at all/Not very much/ Don’t know	56 (56.6)	43 (43.4)	
Somewhat/A great deal	63 (53.4)	55 (46.6)	
Is your decision to be vaccinated influenced by other people’s opinion?			*p* = 0.08
Not at all/Not very much/ Don’t know	88 (51.8)	82 (48.2)	
Somewhat/A great deal	31 (66.0)	16 (34.0)	
Health care provider recommendation			*p* < 0.05
Not at all/Not very much/ Don’t know	19 (41.3)	27 (58.7)	
Somewhat/A great deal	100 (58.5)	71 (41.5)	
Comfort or trust in seeking healthcare			*p* = 0.23
Not at all/Not very much/ Don’t know	17 (45.9)	20 (54.1)	
Somewhat/A great deal	102 (56.7)	78 (43.3)	
**Comfort or trust in healthcare provider**			*p* = 0.68
Not at all/Not very much/ Don’t know	17 (51.5)	16 (48.5)	
Somewhat/A great deal	102 (55.4)	82 (44.6)	
**Self-efficacy**			
Comfort asking health care provider for information about the HPV vaccine			*p* < 0.01
Very uncomfortable/uncomfortable/ unsure	16 (37.2)	27 (62.8)	
Comfortable/Very comfortable	103 (59.2)	71 (40.8)	
Comfort asking health care provider for HPV vaccination			*p* < 0.001
Very uncomfortable/uncomfortable/ unsure	19 (29.2)	46 (70.8)	
Comfortable/Very comfortable	100 (65.8)	52 (34.2)	
**Barriers**			
Vaccine cost			*p* = 0.14
Not at all/Not very much/Don’t know	27 (46.6)	31(53.4)	
Somewhat/A great deal	92 (57.9)	67 (42.1)	
Whether the vaccine is covered by insurance			*p* = 0.80
Not at all/Not very much/Don’t know	21 (56.8)	16 (43.2)	
Somewhat/A great deal	98 (54.4)	82 (45.6)	
Previous negative health care experiences			*p* = 0.06
Not at all/Not very much/Don’t know	54 (62.8)	32 (37.2)	
Somewhat/A great deal	65 (49.6)	66 (50.4)	

*Bold are *p* < 0.10 and qualify for entry into multivariable analysis

In the multivariable analysis, four factors were significantly associated with the intention to obtain an HPV vaccine. Compared with those who did not intend to be vaccinated, those reporting a belief that vaccines are well tested (AOR = 2.28; 95% CI = 1.12–4.63), that they were likely to get an HPV infection (AOR = 2.66; 95%CI = 1.16–6.05), and that they were comfortable asking a health care provider for the HPV vaccine (AOR = 4.53; 95%CI = 1.59–12.88) had greater odds of intending to be vaccinated. In contrast, those reporting that previous negative healthcare experiences would influence their HPV vaccination decision had lower odds of intending to be vaccinated (AOR = 0.40; 95% CI = 0.20–0.80) (Table 4).

**Table 4:** Multivariable analysis: Factors associated with future intent to obtain an HPV Vaccine (RQ #3) (*N* = 207)*

	Adjusted OR (95%CI)
**Characteristic**	
Ever had an abnormal Pap	
No/Don’t know	ref
Yes	2.09 (0.93, 4.67)
Ever had an HPV test	
No/Don’t know	ref
Yes	0.88 (0.43, 1.83)
**Attitudes/beliefs**	
Believe vaccines are well-tested	
No/Don’t know	ref
Yes	2.28 (1.12, 4.63)**
Perceive HPV vaccine to be effective	
Not very effective or DK	ref
Very or fairly effective	1.69 (0.84, 3.41)
Perceived likelihood of getting an HPV infection	
No chance/unlikely	ref
Unsure	1.83 (0.82, 4.07)
Certain/likely/moderately likely	2.66 (1.16, 6.05)**
**Social influences**	
Is your decision to be vaccinated influenced by other people’s opinions?	
Not at all/Not very much/Don’t know	ref
Somewhat/A great deal	2.21 (0.98, 5.01)
Health care provider recommendation	
Not at all/Not very much/Don’t know	ref
Somewhat/A great deal	1.44 (0.58, 3.57)
**Self-efficacy**	
Comfort asking health care provider for information about the HPV vaccine	
Very uncomfortable/ uncomfortable/unsure	ref
Comfortable/Very comfortable	0.52 (0.15, 1.74)
Comfort asking health care provider for information about getting HPV vaccines	
Very uncomfortable/ uncomfortable/unsure	ref
Comfortable/Very comfortable	4.53 (1.59, 12.88)**
**Barriers**	
Previous negative health care experiences	
Not at all/Not very much/Don’t know	ref
Somewhat/A great deal	0.40 (0.20, 0.80)**

* All factors at *p* < 0.10 from bivariate analyses were entered into final model. *P* < 0.05 was considered statistically significant in the final model. The N for the multivariable analysis (207) is lower than the N for the bivariate analyses (217) due to missing values

**Bold are *p* < 0.05

## Discussion

The expansion of eligibility for the HPV vaccine to persons aged 27–45 years provides new opportunities to prevent HPV-related cancers, precancers, and genital warts. However, we found that less than a third of the women in this sample had received one or more doses of the HPV vaccine. Of those who were unvaccinated or unsure about their vaccination history, more than half reported that they were very likely or likely to accept HPV vaccination in the future.

Only a few national studies of mid-adult women (27–45 years) have been conducted since the change in ACIP guidelines [17, 18], though several other studies have looked at both women and men [19, 20]. Our finding that approximately half of those previously unvaccinated were willing to get the vaccine is similar to findings from other national surveys conducted since that time, which ranged from 33% among sexual minority women [21] and 43–54% among broader samples of women [13, 19]. One study found that about 52% of individuals (both women and men) were willing to ask their providers for HPV vaccine information [13]. Studies have also found that perceived vaccine effectiveness, perceived safety [19], perceived vulnerability [19], and perceived likelihood of benefitting from the vaccine [22] were associated with willingness/intent to get the HPV vaccine in this age group. Our study provides new information about the relationship between prior negative healthcare experiences and HPV vaccination. This is notable since those who had negative experiences were 60% less likely to report they would be willing to be vaccinated.

Findings should be viewed in light of study limitations. First, this was a convenience sample from a Qualtrics™ panel, so the findings may not be generalizable to other populations. However, recent research has found that while participants recruited from online panels are not necessarily representative of the U.S. population, they are equivalently representative as traditional recruitment approaches [23]. Regardless, we recognize that those willing to complete research studies may be more likely to be receptive to health interventions than the general public. Additionally, we did not assess HPV vaccine intentions over a specific time frame, as is often done in “stages of change” models. Like most other studies in this field, vaccination status was assessed through self-report, which, while generally accurate among adults, is less accurate than status confirmed via medical records or vaccine registries [24]. We also acknowledge that some of the confidence intervals in our findings were wide. Therefore, findings should be interpreted with caution, as estimates may be unstable. Nevertheless, study strengths include the timeliness of findings vis-a-vis expansion of HPV vaccine eligibility, and the ability to compare across different racial/ethnic groups for whom there has been inadequate representation in prior research.

### Implications for practice

Since SCDM, rather than routine HPV vaccination, is recommended for women in this age group, those not intending to be vaccinated may require no intervention so long as they are fully informed about the potential benefits of vaccination and their individual risk for infection and cervical cancer. However, prior studies indicate that most women do not know that there is now expanded vaccine eligibility [20]. Further, many women, especially those with lower levels of education and who are racially/ethnically minoritized, are not aware that HPV causes cervical cancer [25]. Although we did not assess knowledge of risk factors in this study, our finding that two-thirds of women were willing to be vaccinated and that being comfortable asking one’s healthcare provider for the vaccine suggests that efforts to build awareness and provide skills about engaging in SCDM with one’s provider may hold promise. These efforts will also likely require additional information about the rigor of HPV vaccine testing to promote confidence among some women. Targeted efforts may be needed to engage women who had prior negative experiences in the healthcare system, as they were 60% less likely to report that they would be vaccinated in the future. Existing evidence points to the role of medical mistrust in vaccine hesitancy in general [26], including HPV vaccination [27]. Building trust in the healthcare system will likely necessitate multilevel interventions, not only directed at individuals who have mistrust, but also to enhance trust with providers [28, 29], as well as the trustworthiness of healthcare systems. Comprehensive efforts should also include provider-directed interventions, as the ACIP guidelines do not provide specific information about which patients may most benefit from vaccination. This is especially important since provider recommendation has been identified as the most influential factor in vaccine acceptance [30] and continues to be for this age group (21). Yet, in a recent study of primary care physicians, only 42% had recommended HPV vaccination to adults aged 27–45 years, and 57% were unsure about what to discuss during SCDM conversations [31].

## Conclusion

The expanded eligibility of HPV vaccination for individuals aged 27–45 years has the potential to reduce cervical and other HPV-related cancers, as well as genital warts [32]. However, since a recommendation for SCDM for this age group differs from the routine HPV vaccination recommended for younger age groups, greater efforts to ensure awareness of expanded eligibility and potential benefits may be needed among patients and providers through a mutual discussion about vaccination. Our results suggest that many unvaccinated mid-adult women may be interested in receiving the HPV vaccine, and an even more significant number may be interested once efforts are made to increase their understanding of the safety, efficacy, and testing of the vaccine, as well as expanded eligibility.

### Electronic supplementary material

Below is the link to the electronic supplementary material.

Supplementary Material 1

## Data Availability

The data that support the findings of this study are available from the corresponding author, but restrictions apply to the availability of these data. The data are, however, available from the authors upon reasonable request.
