# Supplementary material for: HPV vaccine behaviors and intentions among a diverse sample of women aged 27-45 years: implications for shared clinical decision-making
Source: BMC Public Health. 2024 Aug 8;24:2154. doi: 10.1186/s12889-024-18740-2 (PMC11308683; doi:10.1186/s12889-024-18740-2)
Supplement: Supplementary file 1 — Supplementary Material 1 [file 12889_2024_18740_MOESM1_ESM.docx]

HPV/Vaccine Survey

Start of Block: Consent

Consent Text The purpose of this research project is to understand knowledge, attitudes and behaviors related to vaccines (e.g., for COVID, HPV). We also ask female participants about cervical cancer screening practices and attitudes. This is a research project being conducted by researchers at Tufts University. You are invited to participate in this research project because you are an adult over the age of 18 years. 


Your participation in this research study is voluntary. You may choose not to participate. If you decide to participate in this research survey, you may withdraw at any time. If you decide not to participate in this study or if you withdraw from participating at any time, you will not be penalized. 


The procedure involves filling out an online survey that will take approximately 15 minutes. Your responses will be anonymous, and we do not collect identifying information such as your name, email address or IP address. The survey questions will be about your knowledge, attitudes and behaviors related to vaccines (e.g., for COVID, HPV). We also ask questions about your health, healthcare, sources of health information, and demographic characteristics. 


We keep your information confidential. All data is stored in a password protected electronic format. To help protect your confidentiality, the surveys will not contain information that will personally identify you. The results of this study will be used for scholarly purposes only and may be shared with researchers from Tufts University.


If you have any questions about the research study, please contact Jennifer Allen (617) 627-0366 or Jennifer.allen@tufts.edu. If you have questions or concerns about your rights as a research participant, or if you would like to discuss the study with someone outside of the research team, contact the Tufts SBER Institutional Review Board (IRB) at 617.627.8804 or sber@tufts.edu.

- I have read the consent statement and agree to participate in the study. (1)
- I do not wish to participate in the study. (2)

Display This Question:

If The purpose of this research project is to understand knowledge, attitudes and behaviors related... = I do not wish to participate in the study.

Q47 Without your consent, you are unable to participate in this study. If you have any questions, please contact the study's primary investigator Jennifer Allen at jennifer.allen@tufts.edu. 
If you believe that you have reached this message in error, please press the back button below to be taken back to the consent information page. Otherwise, please press the next button below to be brought to the end of the survey.

Skip To: End of Survey If Without your consent, you are unable to participate in this study. If you have any questions, ple... Is Displayed

End of Block: Consent

Start of Block: Demographics

Q1 How old are you (in years)?

________________________________________________________________

Q2 Are you of Hispanic, Latino/a, or Spanish origin? Mark all that apply

- No, not of Hispanic, Latino/a, or Spanish origin (1)
- Yes, Mexican, Mexican American, Chicano/a Mexican (2)
- Yes, Puerto Rican (3)
- Yes, Cuban (4)
- Yes, another Hispanic, Latino/a, or Spanish origin (Please specify) (5) ________________________________________________

Q3 What is your race? Mark all that apply

- White (1)
- Black or African American (2)
- Native American, American Indian, or Alaska Native (3)
- Asian Indian (4)
- Chinese (5)
- Filipino/a (6)
- Japanese (7)
- Korean (8)
- Vietnamese (9)
- Another Asian identity (Please specify) (10) ________________________________________________
- Native Hawaiian (11)
- Guamanian or Chamorro Guamanian (12)
- Samoan (13)
- Another Pacific Islander Identity (14) ________________________________________________
- Arab or Arab American (15)
- Something else (16) ________________________________________________

Q4 What is your combined annual income, meaning the total pre-tax income from all sources earned in the past year?

- Less than $20,000 (1)
- $20,000 to $34,000 (2)
- $35,000 to $49,000 (3)
- $50,000 to $74,000 (4)
- $75,000 or more (5)
- Not sure (6)

Q5 What sex were you assigned at birth? (i.e. on your original birth certificate)

- Male (1)
- Female (2)
- Something else (3) ________________________________________________

End of Block: Demographics

Start of Block: Healthcare Coverage

Q6 Do you have any kind of health care coverage, including health insurance, prepaid plans such as HMOs, or government plans such as Medicare or Indian Health Service?

- Yes (1)
- No (2)
- Not sure/Don't know (3)

Display This Question:

If Do you have any kind of health care coverage, including health insurance, prepaid plans such as H... = Yes

Q7 What is the primary source of your health care coverage?

- A plan purchased through an employer or union (including plans purchased through another person's employer) (1)
- A plan that you or another family member buys on your own (2)
- Medicaid or other state program (3)
- Medicare (4)
- TRICARE (formerly CHAMPUS), VA, or Military (5)
- Alaska Native, Indian Health Service, Tribal Health Services (6)
- Some other source (7) ________________________________________________

Q8 Do you have one person you think of as your personal doctor or health care provider?

- Yes (1)
- No (2)
- Not sure/Don't know (3)

Q9 About how long has it been since you last visited a doctor/health care provider for a routine physical exam or check-up?

- A year ago or less (1)
- More than 1 year ago, but less than 2 years ago (2)
- More than 2 years ago, but less than 3 years ago (3)
- More than 3 years ago (4)
- Don't know/Not sure (5)

Q10 Have you delayed or forgone a preventive health care visit (e.g. cancer screening) due to COVID-19?

- Yes (1)
- No (2)
- Not sure/Don't know (3)

End of Block: Healthcare Coverage

Start of Block: All Gender HPV

Q11 Have you ever been told by a healthcare provider that you had or have HPV?

- Yes (1)
- No (2)
- Not sure/Don't know (3)

Q12 Before taking this survey, had you ever heard of the HPV (human papillomavirus) vaccine?

- Yes (1)
- No (2)
- Not sure/Don't know (3)

Skip To: End of Block If Before taking this survey, had you ever heard of the HPV (human papillomavirus) vaccine? = No

Q13 Have you ever had the HPV vaccine?

- Yes (1)
- No (2)
- Not sure/Don't know (3)

Display This Question:

If Have you ever had the HPV vaccine? = Yes

Q14 At approximately what age did you receive the HPV vaccine?

- Under 11 (1)
- 11-14 (2)
- 15-17 (3)
- 18-26 (4)
- 27 or older (5)

Display This Question:

If Have you ever had the HPV vaccine? = Yes

Q15 How many doses did you get?

- 1 (1)
- 2 (2)
- 3 (3)
- Not sure/Don't know (4)

Display This Question:

If Have you ever had the HPV vaccine? = No

Or Have you ever had the HPV vaccine? = Not sure/Don't know

Q16 People age 9-45 are eligible for the HPV vaccine. How likely are you to get the HPV vaccine in the future?

- Very likely (1)
- Somewhat likely (2)
- Somewhat unlikely (3)
- Very unlikely (4)
- Not sure/Don't know (5)

Display This Question:

If People age 9-45 are eligible for the HPV vaccine. How likely are you to get the HPV vaccine in th... = Somewhat unlikely

And People age 9-45 are eligible for the HPV vaccine. How likely are you to get the HPV vaccine in th... = Very unlikely

And People age 9-45 are eligible for the HPV vaccine. How likely are you to get the HPV vaccine in th... = Not sure/Don't know

Q17 If you are unlikely to get the HPV vaccine, please explain

________________________________________________________________

End of Block: All Gender HPV

Start of Block: Women's Health

Q18 A Pap test is a procedure in which a small brush or spatula is used to gently remove cells from the cervix so they can be checked under a microscope for cervical cancer or cell changes that may lead to cervical cancer.
Have you ever had a Pap test?

- Yes (1)
- No (2)
- Not sure/Don't know (3)

Display This Question:

If A Pap test is a procedure in which a small brush or spatula is used to gently remove cells from t... = Yes

Q19 How long has is been since you had your last Pap test?

- Within the past year (anytime less than 12 months ago) (1)
- Within the past 2 years (more than 1 but less than 2 years ago) (2)
- Within the past 3 years (more than 2 years but less than 3 years ago) (3)
- Within the past 5 years (more than 3 years but less than 5 years ago) (4)
- 5 or more years ago (5)
- Not sure/Don't know (6)

Display This Question:

If A Pap test is a procedure in which a small brush or spatula is used to gently remove cells from t... = Yes

Q20 Have you ever had an abnormal Pap test?

- Yes (1)
- No (2)
- Not sure/Don't know (3)

Q21 Have you ever been told by a healthcare provider that you had or have cervical cancer?

- Yes (1)
- No (2)
- Not sure/Don't know (3)

Q22 Have you had a surgery to remove the uterus and cervix (total or radical hysterectomy)?

- Yes (1)
- No (2)
- Not sure/Don't know (3)

End of Block: Women's Health

Start of Block: Self-Swab Questions

Q48 Self-sampling for HPV testing may receive FDA approval for cervical cancer screening within the next year. This would allow patients to collect a vaginal sample either at home or in the clinic to test for HPV (this is done by having the patient insert a soft swab into the vagina).

Q23 If it was an option for you to have a self-swab HPV test, in what location would you want to do it?

- In a doctor's office/clinic (1)
- At home (2)
- Either (3)
- N/A - I would not feel comfortable doing a self-test (4)

Q24-Q26 If this self-swab HPV test became to you, which might be any of your concerns?

|  | Not a Concern (1) | Mild Concern (2) | Moderate Concern (3) | Severe concern (4) |
| --- | --- | --- | --- | --- |
| I want a speculum exam by my health care provider (1) |  |  |  |  |
| I do not trust myself to do a self-test accurately (2) |  |  |  |  |
| I do not feel comfortable with a self-test (3) |  |  |  |  |

Q27-Q32 To what extent do you view the following as potential benefits/advantages of a self-sampled HPV test?

|  | Not a benefit (1) | Small benefit (2) | Moderate benefit (3) | Large benefit (4) |
| --- | --- | --- | --- | --- |
| This test can screen patients for cervical cancer in a clinic where health care providers do not perform speculum exams (1) |  |  |  |  |
| This test can screen patients who have limited geographic access or ability to travel to clinic (live in a remote or rural area, lack transportation) (2) |  |  |  |  |
| This test can screen patients with other logistical barriers (e.g., lack of childcare, inability to secure in-person appointment, etc.)  (3) |  |  |  |  |
| This test would be more convenient/time saving (4) |  |  |  |  |
| This test would be easier for patients who have a history of trauma (5) |  |  |  |  |
| This test would be easier for patients who are uncomfortable with speculum exams for any reason (6) |  |  |  |  |

End of Block: Self-Swab Questions

Start of Block: Perceived Risk of CC

Q33 Compared to the average person your age, would you say that you are...

- More likely to get cervical cancer (1)
- Less likely to get cervical cancer (2)
- At the same risk to get cervical cancer (3)

Q34 How likely do you think it is that you will develop cervical cancer in the next 5 years?

- No chance (1)
- Unlikely (2)
- Moderate chance (3)
- Likely (4)
- Certain (5)
- Don't know (6)

End of Block: Perceived Risk of CC

Start of Block: Flu/COVID Vaccine

Q35 How comfortable are you asking your provider for information about vaccines?

- Very comfortable (1)
- Somewhat comfortable (2)
- Somewhat uncomfortable (3)
- Very uncomfortable (4)
- Don't know/Not sure (5)

Q36 Did you get a flu shot this year?

- Yes (1)
- No (2)
- Not sure/Don't know (3)

Q37 Did you receive (or do you plan to receive) all required doses of the COVID-19 vaccine?

- Yes, I received all required doses (1)
- Yes, I plan to receive all required doses (2)
- No, I have received one dose but do not plan to receive the other (3)
- No, I don't plan to receive any doses (4)

Skip To: Q39 If Did you receive (or do you plan to receive) all required doses of the COVID-19 vaccine? = Yes, I received all required doses

Q38 How likely is it that you will get the COVID-19 vaccine in the future?

- Very likely (1)
- Somewhat likely (2)
- Somewhat unlikely (3)
- Very unlikely (4)
- Don't know/Not sure (5)

Q39 Would you get a COVID-19 booster shot, if recommended?

- Yes (1)
- No (2)
- Not sure/Don't know (3)

Display This Question:

If Did you receive (or do you plan to receive) all required doses of the COVID-19 vaccine? = No, I don't plan to receive any doses

Q40 What is the PRIMARY reason that you do not plan to get a COVID-19 vaccine?

- I am not able to take the vaccine because of a medical condition (1)
- I am not in the group eligible to receive it at this time (2)
- I am not able to get an appointment (3)
- I don't know if a COVID-19 vaccine will work (4)
- I plan to wait until more people are vaccinated to see if it is safe (5)
- I am concerned about possible SHORT TERM side effects of a COVID-19 vaccine (e.g., allergic reactions, pain, fever) (6)
- I am concerned about possible LONG TERM side effects (e.g. fertility, becoming or currently pregnant) (7)
- My doctor has not recommended it (8)
- The vaccine is too expensive (9)
- It’s safer to get the disease than to get the vaccine (10)
- Politics has played too much of a role in the vaccine development process (11)
- The vaccines weren’t tested on people like me (12)
- Other (please specify) (13) ________________________________________________

End of Block: Flu/COVID Vaccine

Start of Block: Vaccine Conspiracy Beliefs

Q41-Q45 To what extent do you agree with the following statements

|  | Strongly agree (1) | Agree (2) | Somewhat agree (3) | Neither agree nor disagree (4) | Somewhat disagree (5) | Disagree (6) | Strongly disagree (7) |
| --- | --- | --- | --- | --- | --- | --- | --- |
| Vaccine safety data is often fabricated (1) |  |  |  |  |  |  |  |
| People are deceived about vaccine effectiveness (2) |  |  |  |  |  |  |  |
| People are deceived about vaccine safety (3) |  |  |  |  |  |  |  |
| Immunizing children is harmful and this fact is covered up (4) |  |  |  |  |  |  |  |
| Pharmaceutical companies cover up the dangers of vaccines (5) |  |  |  |  |  |  |  |

End of Block: Vaccine Conspiracy Beliefs

Start of Block: COVID-19 Social Norms

Q46 Most of my family members have/will get the COVID-19 vaccine

- Strongly agree (1)
- Agree (2)
- Neither agree nor disagree (3)
- Disagree (4)
- Strongly disagree (5)

Q47 Most of my friends have/will get the COVID-19 vaccine

- Strongly agree (1)
- Agree (2)
- Neither agree nor disagree (3)
- Disagree (4)
- Strongly disagree (5)

End of Block: COVID-19 Social Norms

Start of Block: Decision Self-Efficacy

Q48-Q58 How confident do you feel that you can...

|  | Not at all confident (1) | Somewhat confident (2) | Very confident (3) |
| --- | --- | --- | --- |
| Get the information you need to make a decision about getting vaccines (1) |  |  |  |
| Get the facts about the benefits of vaccines (2) |  |  |  |
| Get the facts about the risks of vaccines (3) |  |  |  |
| Understand available information about vaccines enough to be able to make an informed decision about vaccination (4) |  |  |  |
| Ask your health care provider questions about vaccines without feeling dumb. (5) |  |  |  |
| Express your concerns about vaccines to your health care provider. (6) |  |  |  |
| Ask for advice about getting vaccines (7) |  |  |  |
| Figure out the vaccination choices that best suit you personally (8) |  |  |  |
| Handle unwanted pressure from others when making your choice about vaccination (9) |  |  |  |
| Tell your doctor what you think is right for you. (10) |  |  |  |
| Delay your decision if you feel you need more time to make decisions about vaccines (11) |  |  |  |

End of Block: Decision Self-Efficacy

Start of Block: Vaccine Confidence Index

Q59-Q72 Please evaluate how much you disagree or agree with the following statements

|  | Strongly disagree (1) | Disagree (2) | Somewhat disagree (3) | Neither agree nor disagree (4) | Somewhat agree (5) | Agree (6) | Strongly agree (7) |
| --- | --- | --- | --- | --- | --- | --- | --- |
| I am completely confident that vaccines are safe (1) |  |  |  |  |  |  |  |
| Vaccinations are effective. (2) |  |  |  |  |  |  |  |
| Regarding vaccines, I am confident that public authorities decide in the best interest of the community (3) |  |  |  |  |  |  |  |
| Vaccination is unnecessary because vaccine-preventable diseases are not common anymore. (4) |  |  |  |  |  |  |  |
| My immune system is so strong, it also protects me against diseases. (5) |  |  |  |  |  |  |  |
| Vaccine-preventable diseases are not so severe that I should get vaccinated. (6) |  |  |  |  |  |  |  |
| For me, it is inconvenient to receive vaccinations. (7) |  |  |  |  |  |  |  |
| Visiting the doctor’s makes me feel uncomfortable; this keeps me from getting vaccinated. (8) |  |  |  |  |  |  |  |
| When I think about getting vaccinated, I weigh benefits and risks to make the best decision possible. (9) |  |  |  |  |  |  |  |
| For each and every vaccination, I closely consider whether it is useful for me. (10) |  |  |  |  |  |  |  |
| It is important for me to fully understand the topic of vaccination, before I get vaccinated (11) |  |  |  |  |  |  |  |
| When everyone is vaccinated, I don’t have to get vaccinated, too. (12) |  |  |  |  |  |  |  |
| I get vaccinated because I can also protect people with a weaker immune system (13) |  |  |  |  |  |  |  |
| Vaccination is a collective action to prevent the spread of diseases (14) |  |  |  |  |  |  |  |

End of Block: Vaccine Confidence Index

Start of Block: Source of Health Information

Q73-Q78 Below, we ask about places that might provide information about vaccines. Please indicate the extent to which you trust the following entities to provide accurate information about vaccines.

|  | A great deal (1) | Somewhat (2) | Not very much (3) | Not at all (4) | Don't know (5) |
| --- | --- | --- | --- | --- | --- |
| Network news (1) |  |  |  |  |  |
| Social media (2) |  |  |  |  |  |
| Internet (e.g. WebMD, Google) (3) |  |  |  |  |  |
| Public health agencies (e.g. Centers for Disease Control and Prevention) (4) |  |  |  |  |  |
| Governmental agencies (e.g. National Cancer Institute) or officials (5) |  |  |  |  |  |
| Family/Friends (6) |  |  |  |  |  |

End of Block: Source of Health Information

Start of Block: Conjoint Vaccine

| 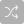 |
| --- |

Q79-Q87 "The following questions each describe a different possible COVID-19 vaccine. Each vaccine specifies how well it works, the side-effects, how many doses are needed, and if booster doses are required. Assume side-effects last 1-3 days. 
 
Assume that all vaccines are safe, approved by the Food and Drug Administration (FDA), and recommended by the Centers for Disease Control (CDC). 
 
**Please mark from 0 to 100 how willing you would be to get each vaccine.**

|  | Extremely likely | Somewhat likely | Neither likely nor unlikely | Somewhat unlikely | Extremely unlikely |
| --- | --- | --- | --- | --- | --- |

|  | 0 | 10 | 20 | 30 | 40 | 50 | 60 | 70 | 80 | 90 | 100 |
| --- | --- | --- | --- | --- | --- | --- | --- | --- | --- | --- | --- |

| This vaccine prevents COVID-19 in 9 out of 10 people who get vaccinated. Side-effects typically include soreness at the injection site and tiredness. It requires 1 dose, and no booster is needed. () | 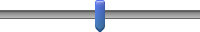 |
| --- | --- |
| This vaccine prevents COVID-19 in 9 out of 10 people who get vaccinated. Side-effects typically include soreness at the injection site and tiredness. It requires 1 dose, and a booster is needed every 10 years. () | 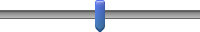 |
| This vaccine prevents COVID-19 in 7 out of 10 people who get vaccinated. Side-effects typically include soreness at the injection site and tiredness. It requires 1 dose, and a booster is needed every 10 years. () | 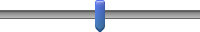 |
| This vaccine prevents COVID-19 in 7 out of 10 people who get vaccinated. Side-effects typically include soreness at the injection site and tiredness. It requires 2 doses, and no booster is needed. () | 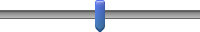 |
| This vaccine prevents COVID-19 in 7 out of 10 people who get vaccinated. Side-effects typically include soreness at the injection site, tiredness, a mild fever, and muscle aches. It requires 1 dose, and a booster is needed every year. () | 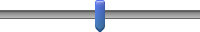 |
| This vaccine prevents COVID-19 in 5 out of 10 people who get vaccinated. Side-effects typically include soreness at the injection site and tiredness. It requires 1 dose, and a booster is needed every year. () | 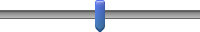 |
| This vaccine prevents COVID-19 in 5 out of 10 people who get vaccinated. Side-effects typically include soreness at the injection site and tiredness. It requires 1 dose, and no booster is needed. () | 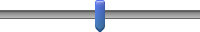 |
| This vaccine prevents COVID-19 in 9 out of 10 people who get vaccinated. Side-effects typically include soreness at the injection site and tiredness. It requires 2 doses, and a booster is needed every year. () | 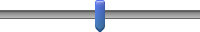 |
| This vaccine prevents COVID-19 in 5 out of 10 people who get vaccinated. Side-effects typically include soreness at the injection site, tiredness, a mild fever, and muscle aches. It requires 2 doses, and a booster is needed every 10 years. () | 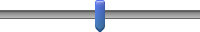 |

End of Block: Conjoint Vaccine

Start of Block: Children Vaccine

Q88 What is the age of your **youngest** child?

- I don't have children (9)
- Under 18 months (1)
- 18 months - 3 years (2)
- 4-6 years (3)
- 7-10 years (4)
- 11-12 years (5)
- 13-15 years (6)
- 16-17 years (7)
- 18 years or older (8)

Skip To: End of Block If What is the age of your youngest child? = I don't have children

Skip To: End of Block If What is the age of your youngest child? = 18 years or older

Skip To: Q90 If What is the age of your youngest child? = 16-17 years

Skip To: Q90 If What is the age of your youngest child? = 13-15 years

Q89 If you have a child (or children) between the ages of 9-12, have they been vaccinated for **HPV**?

- Yes, my child has already received one or more doses of the HPV vaccine (1)
- Yes, I plan to get my child vaccinated for HPV (2)
- No, I don't plan to get my child vaccinated for HPV (3)
- I have multiple children in this age range and I would decide differently for each child (Please explain) (4) ________________________________________________

Q90 If you have a child (or children) 6 months or older, have they been vaccinated for flu this year?

- Yes, my child has already received the flu vaccine this year (1)
- Yes, I plan to get my child vaccinated for the flu this year (2)
- No, I don't plan to get my child vaccinated for the flu this year (3)
- I have multiple children in this age range and I would decide differently for each child (Please explain) (4) ________________________________________________

Q91 If recommended for your child (or children)'s age group, did you or would you have your child vaccinated for COVID-19?

- Yes, my child has already received one or more doses of the COVID-19 vaccine (1)
- Yes, I plan to get my child vaccinated for COVID-19 (2)
- No, I don't plan to get my child vaccinated for COVID-19 (3)
- I have multiple children and I would decide differently for each child (Please explain) (4) ________________________________________________

End of Block: Children Vaccine
